# Hydatid Cyst of the Thigh With Incidental Hepatitis B: A Rare Case

**DOI:** 10.7759/cureus.96444

**Published:** 2025-11-09

**Authors:** Rukayya Usman

**Affiliations:** 1 General Surgery, North Middlesex University Hospital, London, GBR

**Keywords:** albendazole antihelminthic, complex hydatid cyst, echinococcus granulosus (e. granulosus), hepatitis b infection, hepatotoxic drugs, hydatid, intestinal cestode, intramuscular hydatid, musculoskeletal hydatid, treatment choices

## Abstract

Hydatid cyst, also known as cystic echinococcosis, is a zoonotic infection caused by the larval stage of *Echinococcus granulosus*. It primarily affects the liver and lungs, with soft tissue involvement being rare. Coexisting hepatic conditions such as hepatitis B can complicate therapy, as commonly used antihelminthic drugs tend to be hepatotoxic. This report focuses on a 37-year-old woman who presented with a painful swelling in the right thigh after travelling to Bulgaria. Imaging revealed a multiloculated cystic lesion consistent with hydatid disease, confirmed by fine-needle aspiration. She was started on albendazole and praziquantel, but treatment was complicated by an incidental finding of hepatitis B and suspected drug-induced hepatotoxicity. Surgical excision was performed, and she remains under regular multidisciplinary follow-up with MRI surveillance. This case highlights the diagnostic difficulty of extrahepatic hydatid disease and the importance of individualised management when liver comorbidities restrict antiparasitic therapy.

## Introduction

Hydatid disease, also known as echinococcosis, is caused by the *Echinococcus* species. In *Echinococcus granulosus* (*E. granulosus*), humans are infected by ingesting the eggs of the adult *E. granulosus* cestode, which are then excreted in faeces by definitive hosts, typically dogs or other canids. This results in the release of oncospheres into the intestine, which subsequently develop into cysts. *E. granulosus* is distributed worldwide; however, this organism is endemic to the former Soviet Union and Eastern Europe, North Africa, South America, and Central Asia [1]. Hydatid disease can remain dormant for many years until the cysts become large enough to cause symptoms that vary depending on location ​[2]. The organs most affected are the liver (68.8-80%) and lungs (10-22.4%), with musculoskeletal involvement being rare (3%) ​[3]. As such, these uncommon localisations can cause diagnostic challenges for clinicians, often leading to delayed treatment and inappropriate initial management ​[4]. Differential diagnoses, particularly for musculoskeletal hydatids, can include abscesses, soft tissue tumours, osteomyelitis, and lytic bone lesions [5,6]. Management is split into medical therapy with chemotherapeutic drugs primarily from the benzimidazole family and surgery, and in most cases, these are combined for maximum efficacy [7]. This poses a challenge for clinicians in patients with coexisting hepatic conditions such as hepatitis B, as benzimidazoles are known to cause hepatotoxicity.

Herein, we report a rare case of a hydatid cyst in the fascia of the thigh of a 37-year-old female patient, further complicated by an incidental finding of hepatitis B. This report highlights the importance of an individualised treatment plan for such complex patients in which comorbidities may contraindicate anti-helminthic chemotherapy.

## Case presentation

A 37-year-old woman, residing in London, United Kingdom, presented to the emergency department with a one-month history of progressively worsening pain and swelling over the right thigh following a recent trip back from Bulgaria. The patient denied direct contact with dogs or such canids; however, she recalled an instance a few months prior in which she ate unwashed cherries from a market.

**Figure 1:**
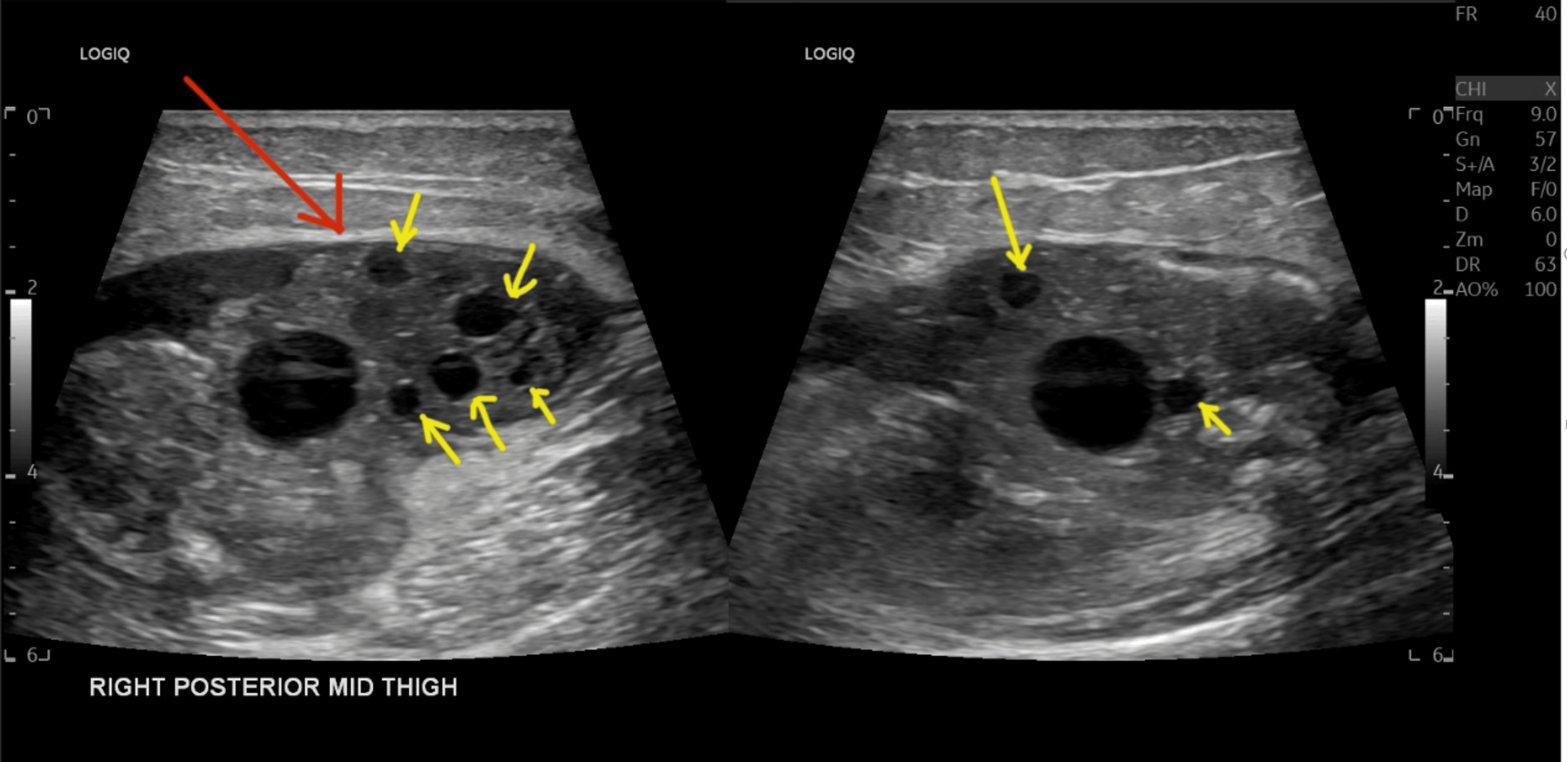
USS Doppler of the right leg (veins) A large, calcified, echogenic, and echocomplex subcutaneous mass in the right posterior mid-thigh, measuring approximately 9 × 5 × 8 cm with a cystic and solid component (red arrow). Multiple well-defined avascular cysts of varying sizes within this mass, representing daughter cysts (yellow arrow).

**Figure 2:**
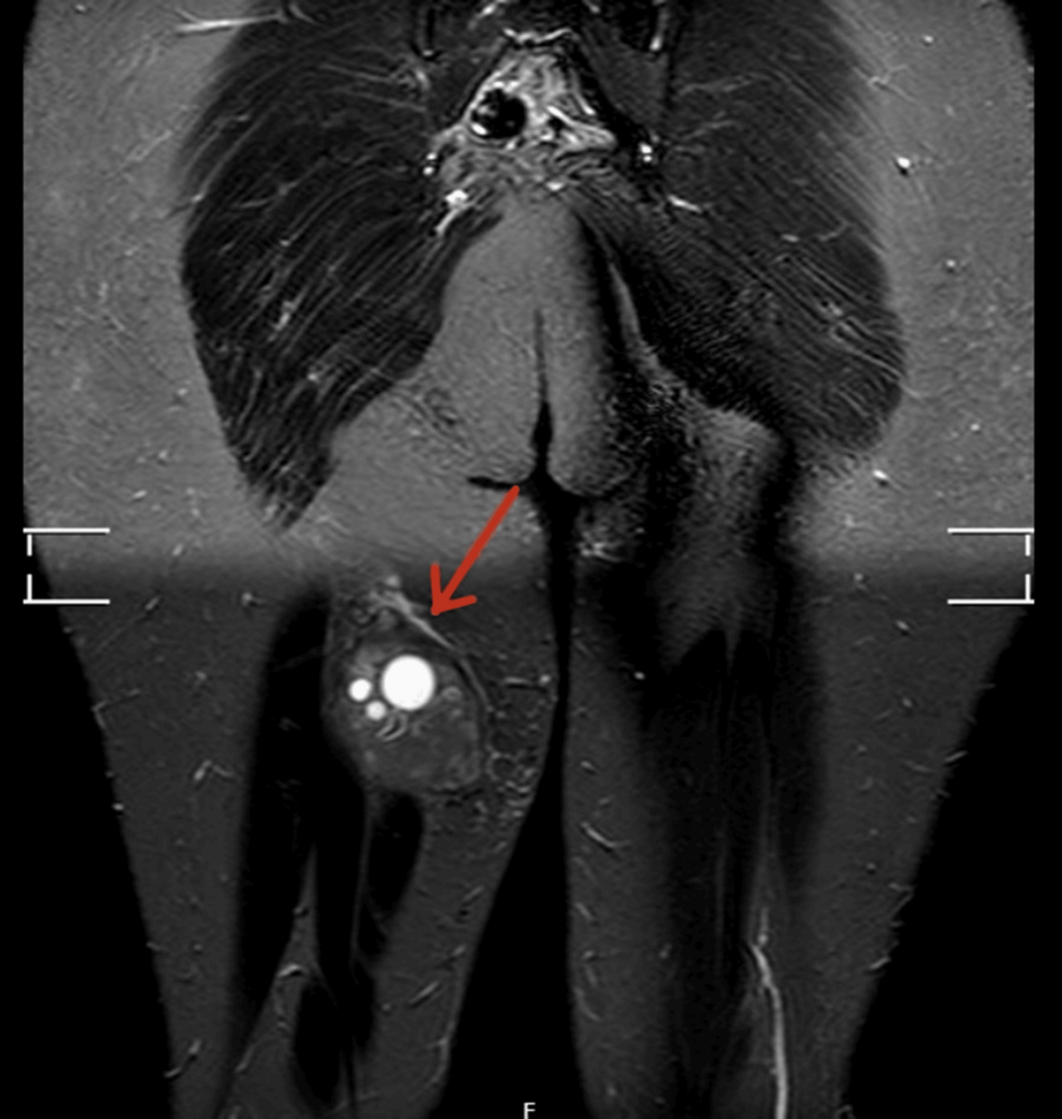
Coronal MRI with contrast of the pelvis and proximal thighs (lesion indicated in the right thigh soft tissue) Coronal contrast-enhanced STIR-MRI demonstrates a 3.8 × 6.2 × 6.8 cm (AP × TS × CC) complex cystic lesion (red arrow) in the posterior compartment of the mid-right thigh. The lesion shows peripheral wall enhancement, which is slightly thickened anteroinferiorly, and causes indentation of the semimembranosus muscle. Multiple small internal cysts (“daughter cysts”) are seen within the main cystic cavity, consistent with a multiloculated hydatid cyst. STIR: short tau inversion recovery

**Figure 3:**
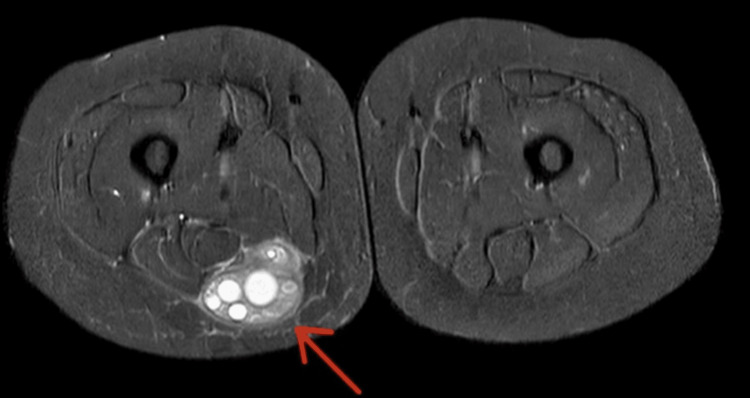
Axial MRI with contrast of the right thigh Axial contrast-enhanced STIR MRI showing the multiloculated cystic lesion (red arrow) located posterior to the semimembranosus tendon and partially extending into the proximal semimembranosus muscle. The lesion lies subfascially, bowing the superficial fascia of the posterior compartment. Peripheral post-contrast enhancement of the cyst wall and septations is evident, with mild perilesional soft-tissue oedema. STIR: short tau inversion recovery

**Figure 4:**
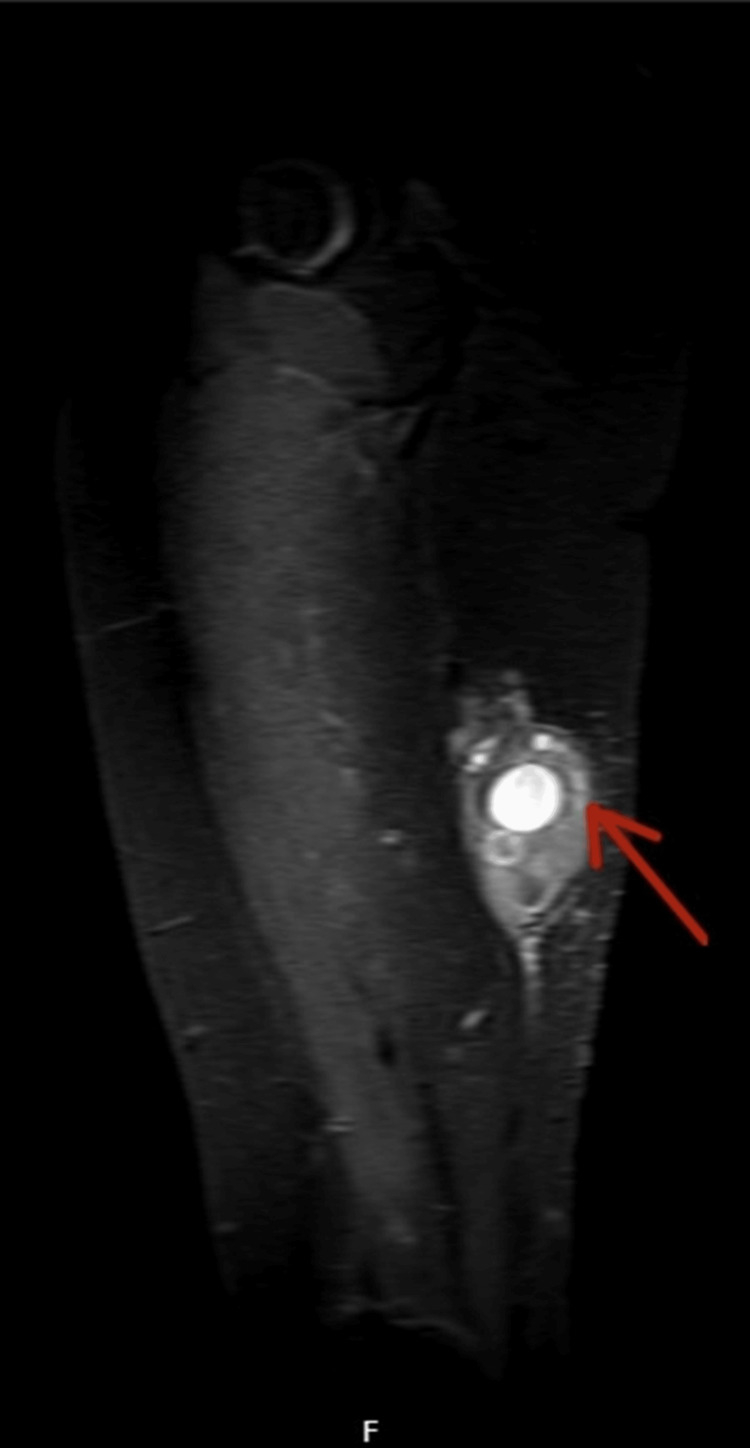
Sagittal MRI with contrast of the right thigh Sagittal contrast-enhanced STIR MRI demonstrating the multiloculated cystic lesion (red arrow) containing multiple daughter cysts with enhancing outer wall and non-enhancing internal cysts. The lesion is predominantly posterior to the semimembranosus, semitendinosus, and biceps femoris muscles, partially involving the proximal semimembranosus. Minimal intramuscular oedema is noted. STIR: short tau inversion recovery

On examination, a large swelling was noted on the posterior aspect, which was tender to the touch and had intact skin overlying the mass. There was no generalised leg swelling, skin discolouration, dilated veins, or warm skin. The remainder of the physical exam was unremarkable. Due to the recent travel and elevated D-dimer, emergency care clinicians requested a Doppler ultrasound (USS) of the right leg veins to rule out deep vein thrombosis (DVT). USS ruled out DVT; however, it revealed a large, right posterior thigh subcutaneous calcified mass measuring 9 x 5 x 8cm with multiple cysts of varying sizes, reported as highly suspicious for hydatid disease (Figure 1). The patient was then referred to our team, and an MRI revealed a 3.8 x 6.2 x 6.8 cm complex cystic lesion in the right mid-thigh posterior compartment, located in the subfascia and indenting the semimembranosus muscle (Figures 2-4).

An USS-guided fine needle aspiration biopsy confirmed* E. granulosus*, with the cyst classified as CE3B according to the World Health Organisation Informal Working Group on Echinococcosis (WHO-IWGE) classification of cystic echinococcosis. With the input of the infectious disease and parasitology team, the patient was started on 200mg of albendazole twice daily (BD) and 400mg of praziquantel once daily (OD), and was scheduled to have the lesion resected. 

**Figure 5:**
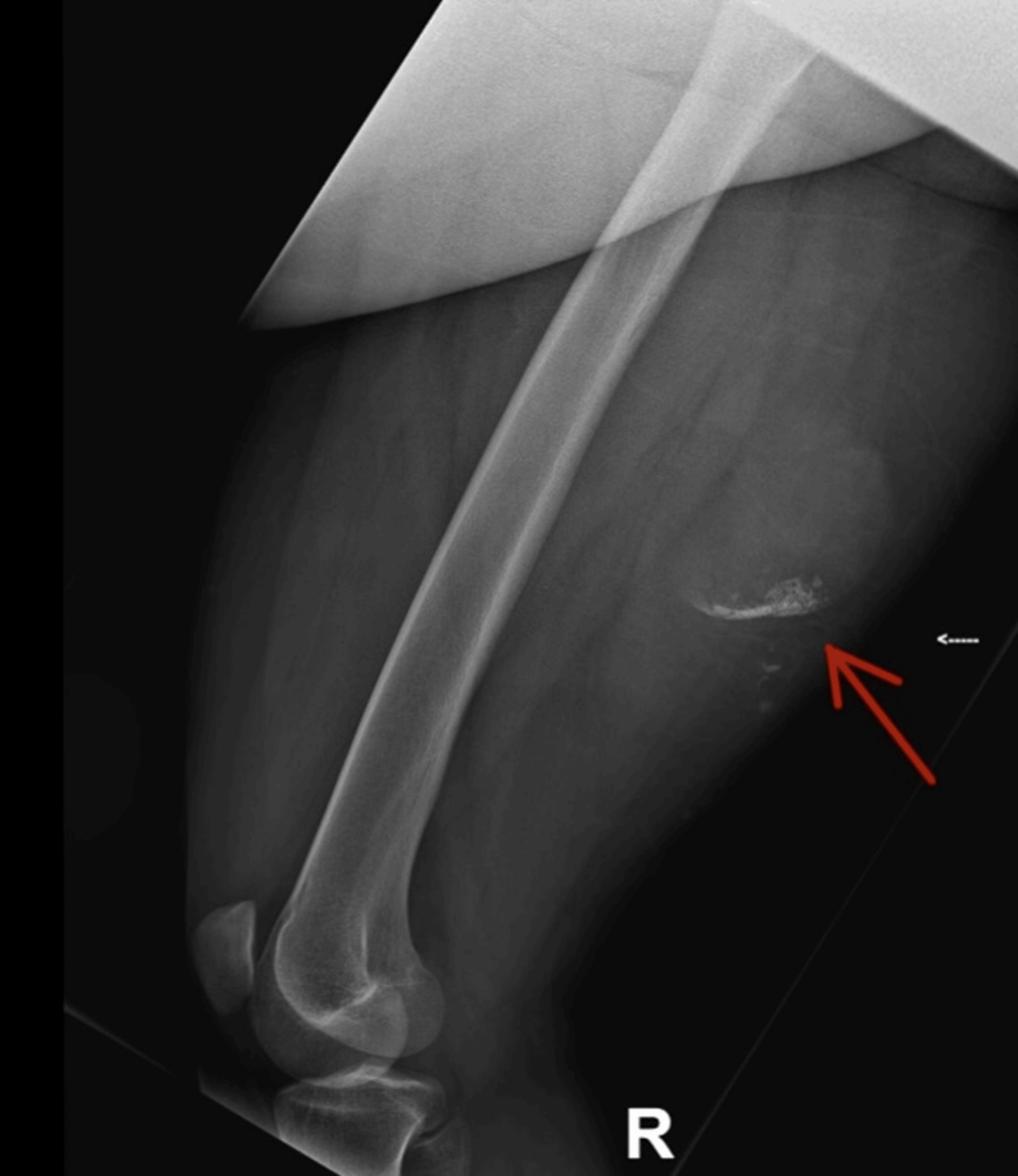
Lateral X-ray of the right femur Patchy punctate consolidation within a poorly seen soft tissue opacity projected at the posteromedial aspect of the mid-distal thigh in keeping with the suspected hydatid cyst. No signs of osteomyelitis.

**Figure 6:**
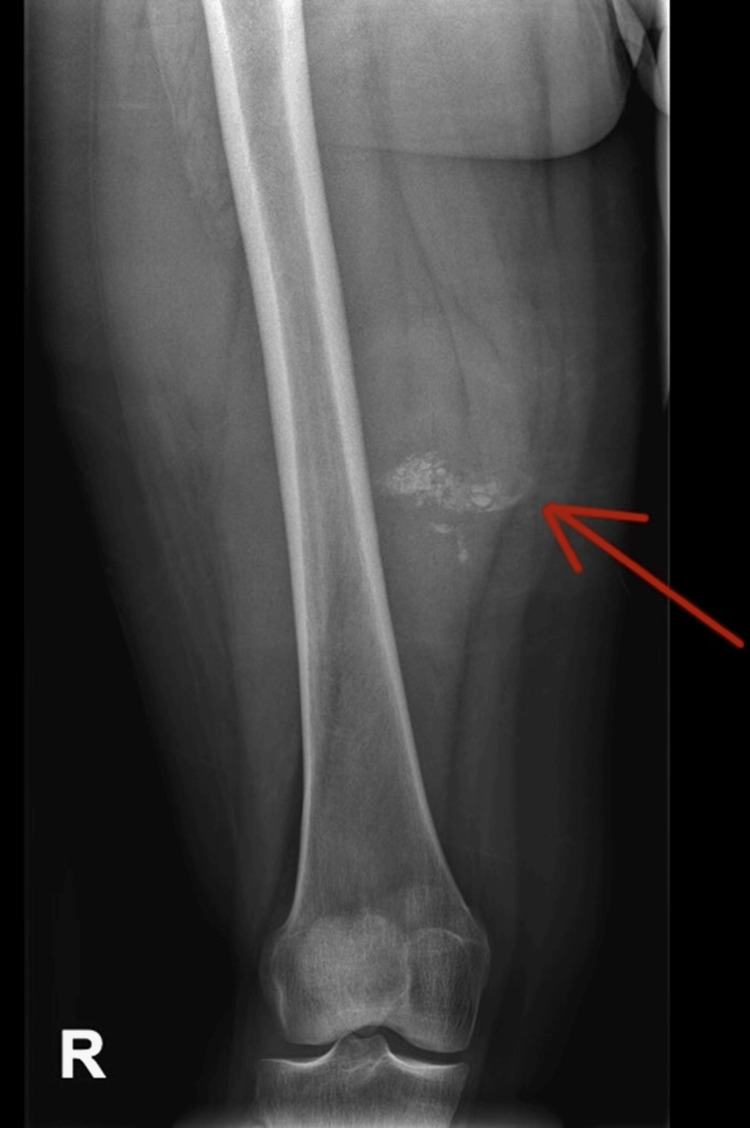
Anteroposterior (AP) X-ray of the right femur Patchy punctate consolidation within a poorly seen soft tissue opacity projected at the posteromedial aspect of the mid-distal thigh in keeping with the suspected hydatid cyst. No signs of osteomyelitis.

Unfortunately, a month later, this patient re-presented to the emergency department with superadded bacterial infection at the biopsy site, requiring hospital admission. Interestingly, this time, clinicians believed the lesion was a large sebaceous cyst that had progressed to osteomyelitis; hence, the patient underwent an X-ray (Figures 5-6). She was started on ceftriaxone for a wound culture positive for heavy growth of *Staphylococcus aureus*.   

It was during this admission that the patient presented with new symptoms of severe headache with blurred vision, right upper quadrant, as well as epigastric abdominal pain. Further scans were organised with the fear of neurocysticercosis and/ or spread of the cysts to other viscera. CT head scan revealed no calcified or cystic lesions, and no acute intracranial pathology was identified. CT chest, abdomen, and pelvis + contrast confirmed no hepatic or pulmonary hydatid disease, both common primary sites of echinococcosis. This, however, showed hepatomegaly and enlarged paraportal lymph nodes, consistent with a liver infection.

Hepatitis B surface antigen (HBsAG) and hepatitis B viral DNA (HBV DNA) came back elevated, confirming hepatitis B with transaminitis and no features of hepatic decompensation. Thus, she was started on tenofovir 245mg OD. Once discharged, the patient was planned to undergo resection in the UK; however, she returned to her home country, Bulgaria, to have the operation done there.  In Bulgaria, she was discharged with and completed  a short six-week course of albendazole without additional praziquantel, as this was stopped earlier by the patient due to associated headaches.    

Upon her return to the UK, further doses of albendazole were suspended as she was thought to have albendazole-induced hepatitis on a background of elevated ALT secondary to chronic hepatitis B. The follow-up plan post-resection included careful monitoring over the next three years, with an MRI of the thigh every six months. If there is no evidence of recurrence after three years, the frequency of scans should be changed to yearly for a total of 10 years. Patient compliance was emphasised, as it is easier to manage a hydatid cyst recurrence if caught early. The patient remains under long-term monitoring by the hepatology team, as well as the parasitology and infectious disease team.    

## Discussion

Hydatid disease (echinococcosis) is usually caused by the larval stage of *E. granulosus*, which produces unilocular cystic lesions. They are generally slow-growing and remain asymptomatic until the involved organ elicits symptoms  ​[8]. Clinical symptoms are often non-specific, causing diagnosis to be challenging, such as the case of our patient, whose initial differential diagnoses included DVT and osteomyelitis. The adult tapeworm is found in the small intestine of the canine (definitive host), and it in turn passes eggs in their faeces, which are accidentally ingested by humans (intermediate hosts). The larva hatches from the egg, penetrating the intestinal lining and entering the bloodstream, which then disseminates to any organ, with the liver and lungs being the most frequently involved. It develops into a hydatid cyst. Hydatid disease in the muscle is rare due to the high content of lactic acid in the muscle, making it an unsuitable environment for parasitic growth due to the lack of oxygen ​[9,10].

Imaging is highly dependent on the organ involved, with MRI being the gold standard in soft tissue involvement [11]. For example, X-rays can define pulmonary cysts; however, they may miss other cysts in other organs unless there is cyst wall calcification. The pathognomonic finding on CT or MRI is the presence of daughter cysts within the larger cyst, along with eggshell or mural calcification, which indicates *E. granulosus* invasion and helps distinguish it from other pathologies commonly mistaken for it, such as abscesses. An accurate diagnosis can be made by fine-needle aspiration of fluid and examination for adult *E. granulosus *scoliceal hooklets ​[12]. However, this is generally not recommended due to the risk of fluid leakage, which could potentially result in dissemination or anaphylactic reactions.    

Treatment of hydatid disease takes into consideration the location, size, and manifestations of cysts in the patient. The World Health Organisation (WHO) provides guidelines, but there is no single standard that all clinicians should follow regarding the duration, choice of treatment, or dose of chemotherapy drugs [13]. The medical therapy consists of chemotherapy drugs primarily from the benzimidazole family, in particular, albendazole. Medical therapy can be trialled alone or in combination with surgical resection or aspiration. The definitive management is surgery, and it is indicated for complex lesions, such as those compressing vital structures, large cysts, or cysts with daughter cysts, as in this case. But, this does not come without the risk of fluid leakage during surgery, which can lead to anaphylaxis and the dissemination of infected scolices. Although this complication has been greatly minimised through the use of scolicidal solutions, such as hypertonic saline or ethanol ​[14]. Pre- and post-surgical resection use of benzimidazoles is often combined to achieve the best efficacy ​[7], though surgery can eliminate the need for medical therapy ​[15]. 

In our case, medical therapy was complicated by the fact that the patient developed albendazole-induced hepatotoxicity on a background of chronic hepatitis B. A decision was made to discontinue albendazole and continue tenofovir post-resection following MDT discussions with hepatology, gastroenterology, infectious disease, and parasitology. We were only able to find one other published report detailing hydatid disease co-existing with viral hepatitis, where albendazole-induced hepatotoxicity necessitated therapy cessation ​[16]. This aligns with our patient’s course, demonstrating how liver comorbidity can restrict benzimidazole use. This case highlights the significance of surgery being the definitive treatment for hydatid disease, particularly in patients with complex lesions or concomitant liver disease. Providing the cyst is excised completely and there are no complications, surgery can be sufficient without the need for adjuvant therapy ​[17,18].

 As in any case of hydatid disease, irrespective of the management choice, response to treatment and monitoring for recurrence are assessed by serial evaluation using MRI or CT. In cases where medical therapy is contraindicated, it is crucial to detect recurrence early and manage it before the cyst becomes symptomatic.    

## Conclusions

Hydatid disease is a zoonotic infection whose diagnosis remains challenging for clinicians, as symptoms are non-specific and based on the organs involved. High clinical suspicion is required, and it is largely aided by diagnostic imaging, especially in rare extrahepatic locations, such as the musculoskeletal system. Diagnostic methods such as fine needle aspiration, whilst accurate, can pose complication risks which clinicians should keep in mind. Management is patient-specific and must consider comorbidities such as liver pathologies that can be exacerbated by hepatotoxic benzimidazoles. Surgical excision remains the mainstay treatment in such patients and is often sufficient without the need for adjuvant therapy. Ultimately, the use of a multidisciplinary team approach is crucial in managing such complex patients.
